# Socio-demographic and lifestyle factors associated with intrinsic capacity among older adults: evidence from India

**DOI:** 10.1186/s12877-022-03558-7

**Published:** 2022-11-12

**Authors:** K. Muneera, T. Muhammad, S Althaf

**Affiliations:** 1 National Institute of Technology, Calicut, 673601 Kerala India; 2 International Institute for Population Sciences, Mumbai, Maharashtra 400088 India

**Keywords:** Intrinsic capacity, Lifestyle factors, Older adults, India

## Abstract

**Background:**

Since the traditional models missed the possibility of formulating personalised programs centred on a person’s priorities and values, it was a pressing priority to shift from traditional disease-centred to a function-based approach of healthy ageing, which is defined as ‘the process of developing and maintaining the functional ability that enables well-being in older age’. The present study aimed to assess the prevalence of high intrinsic capacity (IC) of older adults and to examine the socio-demographic and lifestyle factors associated with IC among older adults in India.

**Methods:**

The study utilises the individual-level data from the first wave of the Longitudinal Aging Study in India (LASI) conducted during 2017–18. The total sample size for the present study was 24,136 older adults (11,871 males and 12,265 females) aged 60 years and above. Descriptive statistics, along with bivariate analysis, was employed to present the preliminary results. Additionally, multivariable linear and logistic regression analyses were conducted to find out the association of socio-demographic and lifestyle factors with IC and its components.

**Results:**

The mean IC score was found to be 7.37 (SD = 1.6) in this study. A proportion of 24.56% of older adults was observed to be in the higher IC category. Increasing age was negatively associated with high IC for older men and women. Older people who smoke tobacco (β = -0.23; CI: -0.32—-0.13) and chew tobacco (β = -0.11; CI: -0.18—-0.03) were less likely to experience high IC compared to their respective counterparts. Older adults who reported episodic alcohol drinking were less likely to have high IC (β = -0.20; CI:-0.32—-0.07). The engagement in moderate physical activity (β = 0.12; CI:0.01–0.23), vigorous physical activity (β = 0.12; CI:0.05–0.20) and yoga-related activity (β = 0.18; CI:0.09–0.26) were significantly positively associated with high IC. Among the five domains of IC, education was significantly associated with higher capacity in each domain, and increasing age was found to be a significant predictor of lower capacity in each IC domain except locomotion. Older men and women engaged in vigorous physical activity had 35 and 19% significantly higher odds of high capacity in sensory (aOR = 1.35; CI: 1.12—1.62) and psychological (aOR = 1.19; CI: 1.06—1.34) domains, respectively.

**Conclusions:**

The study revealed that lifestyle behaviours including tobacco use, episodic alcohol drinking and physical activity are strongly associated with IC among older adults in India. The findings suggest that healthy lifestyle behaviours should be encouraged among older adults as an effort to improve their IC, which is the key determinant of functional ability and quality of life in later years of life.

**Supplementary Information:**

The online version contains supplementary material available at 10.1186/s12877-022-03558-7.

## Background

Older adults are a rapidly growing fraction of the population worldwide [1]. The number of older adults is expected to grow exponentially to over 1.5 billion in 2050 [2]. Therefore, it is important for older adults to protect their rights to health and enjoy a good quality of life as they age. As a response to population ageing, different conceptual models such as active ageing, successful ageing or healthy ageing have been proposed to address the notion of ageing well [3]. But all these models focused on the absence of clinical diseases or the accumulation of deficits. These traditional disease-centred approaches were inadequate to address the emerging needs of the older population [4]. Since the traditional models missed the possibility of formulating personalised programs centred on a person’s priorities and values, it was a pressing priority to shift from traditional disease-centred to a function-based approach of healthy ageing, which is defined as ‘the process of developing and maintaining the functional ability that enables well-being in older age’.

In 2015, World Health Organization (WHO) introduced the novel construct of intrinsic capacity (IC) to define healthy ageing [5]. The IC refers to an individual’s physical and mental capacities that determine the functional ability of that person, which is the core of healthy ageing [1]. The introduction of the IC construct is focused at measuring the capacities rather than deficits of multiple human biological systems based on body functions which are most relevant to healthy ageing [6]. It is envisaged that a new function-based approach will help to detect geriatric conditions such as person’s fragility, care dependence and disability at the earlier stage and create opportunities for delaying and preventing such conditions. Moreover, IC has been quantified and a probable structure proposed, and it has been shown to be a strong predictor of care dependence and other health outcomes in late life [6–9].

It is relevant to identify various socio-demographic elements and modifiable risk factors which influence IC. Thus, IC can be promoted to delay or prevent adverse health events by focusing on such elements and factors. Some of the earlier studies have investigated the role of socio-demographic factors on IC [10–15]. Significantly lower IC scores were reported for older age groups, women [11, 16], and those with lower levels of education and subjective social status [8]. IC was found to be a potent mediator for the effect of age, sex, and wealth on care dependence [7]. Along with a number of correlates, northern residence and being unmarried were associated with a decline in IC in a recent Chinese study [14]. There is a lack of studies examining the impact of work status on IC, and only one study identified labour market participation as a covariate and reported a non-significant association with IC level [13]. A couple of studies have captured the positive impact of socioeconomic status on IC scores [9, 11, 14]. Household size and household consumption per capita in quantile are considered proxy measurements of socioeconomic status [14]. A few studies explained morbidity [16–18] and polypharmacy [12, 16, 19] as predictors of IC.

Healthy lifestyle behaviours have been consistently associated with better health outcomes in older people [20] and increase in life years spent in good health [21]. Only a few research have looked into the impact of lifestyle behaviours on IC. Mainly, smoking, drinking, physical activity(exercise), diet and nutrition intake are considered health risk behaviour factors in ageing studies. It has been observed that paying attention to one's lifestyle, such as food, exercise, not smoking or drinking excessively, and remaining mentally active, are the most effective approach to healthy ageing [22]. In the IC context, a study examined the association between dietary patterns and IC and its sub-domains in Chinese community-dwelling older adults and explored that various dietary patterns were associated with greater IC and its sub-domains in Chinese community-dwelling older adults, and more associations were observed in men than women [23]. It is also evident that less exercise and less meat intake were related to IC decline [14]. People engaging in physical activity had increased odds of higher IC and thereby enjoying healthy ageing [14, 16, 19].

Globally, studies on the IC are at the nascent stage. Currently, available studies are conducted only in a few countries. Studies conducted in low- and middle-income countries except China are scarce. Since IC is a better predictor of health outcomes [12] and a proxy measure of the quality of life [24], a country like India, witnessing rapid growth of the aged population and seeking for appropriate strategies to cope with the ageing population urgently demands studies focusing on IC which can better predict health outcome and thereby guide the development of personalised interventions which ensure the quality life of older adults. The present study aimed to compute a composite IC score in the Indian context and to examine the socio-demographic and lifestyle factors associated with IC among older adults in India. This study hypothesised that healthy lifestyle behaviours such as non-drinking, non-smoking and engagement in physical activities are positively associated with the higher IC of older adults.

## Methods

### Data

The present study utilises the individual-level data from the first wave of the Longitudinal Aging Study in India (LASI) conducted during 2017–18. The LASI is a country-representative longitudinal survey of more than 72,000 adults aged 45 years and over across all states and union territories of the country that provides vital information on the social, physical, psychological, and cognitive health of the Indian aging population. The LASI survey was conducted through a partnership of the International Institute for Population Sciences (IIPS), Harvard T. H. Chan School of Public Health (HSPH), and the University of Southern California (USC). In the LASI wave 1, the sample selection is based on a multistage stratified cluster sample design, including a three-stage sampling design in rural areas and a four-stage sampling design in urban areas. The details of sampling design, survey instruments, and data collection procedures are provided elsewhere. The present study is conducted on eligible respondents aged 60 years and above. Thus, the total sample size for the present study after dropping the missing observations of the outcome variable (n = 7,328) was 24,136 older adults (11,871 males and 12,265 females) aged 60 years and above.

### Measures

#### Outcome variable

Intrinsic capacity (IC) as the outcome variable of this study was assessed using the criteria summarised in Table 1.

**Table 1:** Assessment of IC among older adults

**Domains** (scoring)	**Measures**	**Description**
**Cognition (0–27 scale)** (recoded as 0 if scored 0–6, considered as cognitively impaired or demented, 1 if scored 7–11 considered as mild cognitive impairment and 2 if scored 12–27 considered as normal) [25]	Episodic memory	Based on Immediate word recall and delayed word recall. Immediate word recall refers to total number of words recalled from the given list of 10 words immediately. Delayed word recall refers to total number of words recalled from the same given list of 10 words after some delay (0–20 points)
	Working memory	Serial seven subtraction (0–5 points)
	Attention	Backward counting (0–2 points)
**Locomotion** (recoded as 0, both gait and balance impaired, 1, either impaired and 2 neither impaired) [26]	Walking speed/Gait speed (Measure of functional capacity)	Time taken to walk a 4 m distance at usual pace
	Standing balance (Indicator of static balance, measured progressively from semi-tandem to either side-by-side or full tandem)	Test of standing balance that progressively gets more difficult (side-by-side stand, semi-tandem, full-tandem)
**Sensory (**recoded as 0, both vision and hearing impaired, 1, either impaired and 2 neither impaired**)**	Distance/ Near vision	Participants were asked to rate “How good is your eyesight for seeing things at a distance/ up close” & Diagnosed with eye or vision problem
	Hearing	Participants were asked regarding diagnosis of hearing related problem
**Vitality (**recoded as 0 if lower BMI/underweight, 1, normal weight and 2 higher BMI/overweight or obese**)** [14]	BMI (Indicator of obesity and the balance between energy intake and energy expenditure)	Body Mass Index (BMI) refers to weight in kilograms divided by height in meter square (kg/m2). BMI levels have been classified according to WHO classifications: underweight ≤ 18.4; normal = 18.5 to 24.9; overweight = 25.0 to 29.9; obese ≥ 30.0
**Psychological (0–30 scale) (**recoded as 0 if scored 20–30, considered as severe symptoms, 1 if scored 10–20, considered as mild symptoms and 2 if scored 0–10, considered as no/minimal depression symptoms**)**	The Centre for Epidemiological Scale of Depression (CES-D) scale	Self-report depression scale of CES-D with a score ranging from 0 to 30, the higher score representing higher depressive symptoms

Each of the domain was assigned a score of 0, 1 or 2, as shown in Table 1, and we summed all five domains to derive a composite score of IC ranging from 0 to 10, with higher scores representing greater IC. Further, a recent study showed that every standard deviation increment in the mean IC composite score was associated with better functional ability [27]. Similarly, in this study, one standard deviation increment from the mean IC score was considered as the cut-off score for high IC among older adults during the bivariate analysis. The continuous scale of IC ranging from 0 to 10 was used in the multivariable analyses in this study.

#### Covariates

Socio-demographic variables included age (recoded as 60–69, 70–79 and 80 +), sex (male and female), education (recoded as none, primary, secondary and higher), marital status (recoded as married, widowed and others which included separated, divorced and never married) and work status (recoded as never worked, currently working, not working and retired).

The current tobacco smoking was sourced from the item (1) “Do you currently smoke any tobacco products (cigarettes, *bidis*, cigars, hookah, cheroot, etc.)?” and current smokeless tobacco use was sourced from the item (2) “Do you use smokeless tobacco (such as chewing tobacco, *gutka*, *pan masala*, etc.)?” The variables were dichotomised to yes and no, representing the current use of smoked and smokeless tobacco. Similarly, episodic alcohol drinking use was assessed with the question, “In the last 3 months, how frequently, on average, have you had at least 5 or more drinks on one occasion?”, and defined as yes if the response was “one to three days per month, one to four days per week, five or more days per week, or daily”. Moderate physical activity was measured through the question, “How often do you do activities such as cleaning house, washing clothes by hand, fetching water or wood, drawing water from a well, gardening, bicycling at a regular pace, walking at a moderate pace, dancing, floor or stretching exercises? Similarly, the question through which vigorous physical activity was assessed was “How often do you take part in sports or vigorous activities, such as running or jogging, swimming, going to a health centre or gym, cycling, or digging with a spade or shovel, heavy lifting, chopping, farm work, fast bicycling, cycling with loads”? Both the variables were dichotomised to yes (every day, more than once a week, once a week, one to three times in a month), and no (hardly ever or never). The yoga-related activity was assessed using the question, How often do you engage in the activities such as yoga, meditation, asana, pranayama or similar? The variable was dichotomised as yes (every day, more than once a week, once a week, one to three times in a month), and no (hardly ever or never).

Further, the monthly per capita consumption expenditure (MPCE) quintile was assessed using household consumption data. The details of the measure are published elsewhere. The variable was then divided into five quintiles i.e., from poorest to richest. Religion was coded as Hindu, Muslim and Others. Caste was recoded as Scheduled Caste/Scheduled Tribe (SC/ST), Other Backward Classes (OBC), and others. The other caste category refers to those having higher social status, mostly belonging to upper caste categories. The place of residence was coded as urban and rural. Also, the regions of the country were coded as North, Central, East, Northeast, West, and South.

#### Statistical analysis

Descriptive statistics, along with results of cross-tabulations, were reported in the study. We checked whether the IC followed a normal distribution by visual inspection with a histogram. We also computed Pearson's correlation coefficient between IC and age to explore the relationship between these measures.

Additionally, multivariable linear regression for IC score and multivariable binary logistic regressions for each component was conducted to find the association of socio-demographic and lifestyle factors with IC and its components. The estimates were presented in the form of adjusted coefficients (aCoef) and adjusted odds ratios (aOR) with a 95% confidence interval (CI). All statistical models were adjusted for the selected background characteristics, including age, sex, education, marital status, work status, smoked and smokeless tobacco use and alcohol consumption, moderate and vigorous physical activity, yoga-related activity, MPCE quintiles, religion, caste, place of residence and regions.

The statistical analysis was performed using Stata 15.1. Survey weights were applied to account for the population level estimates. Regression diagnostics included tests for multicollinearity of predictor variables and tests for normality of residuals with variance inflation factor (VIF) and kernel density plots (Supplementary material Table S1 and Figure S1) and found no violations of basic assumptions for regression.

## Results

**Fig. 1:**
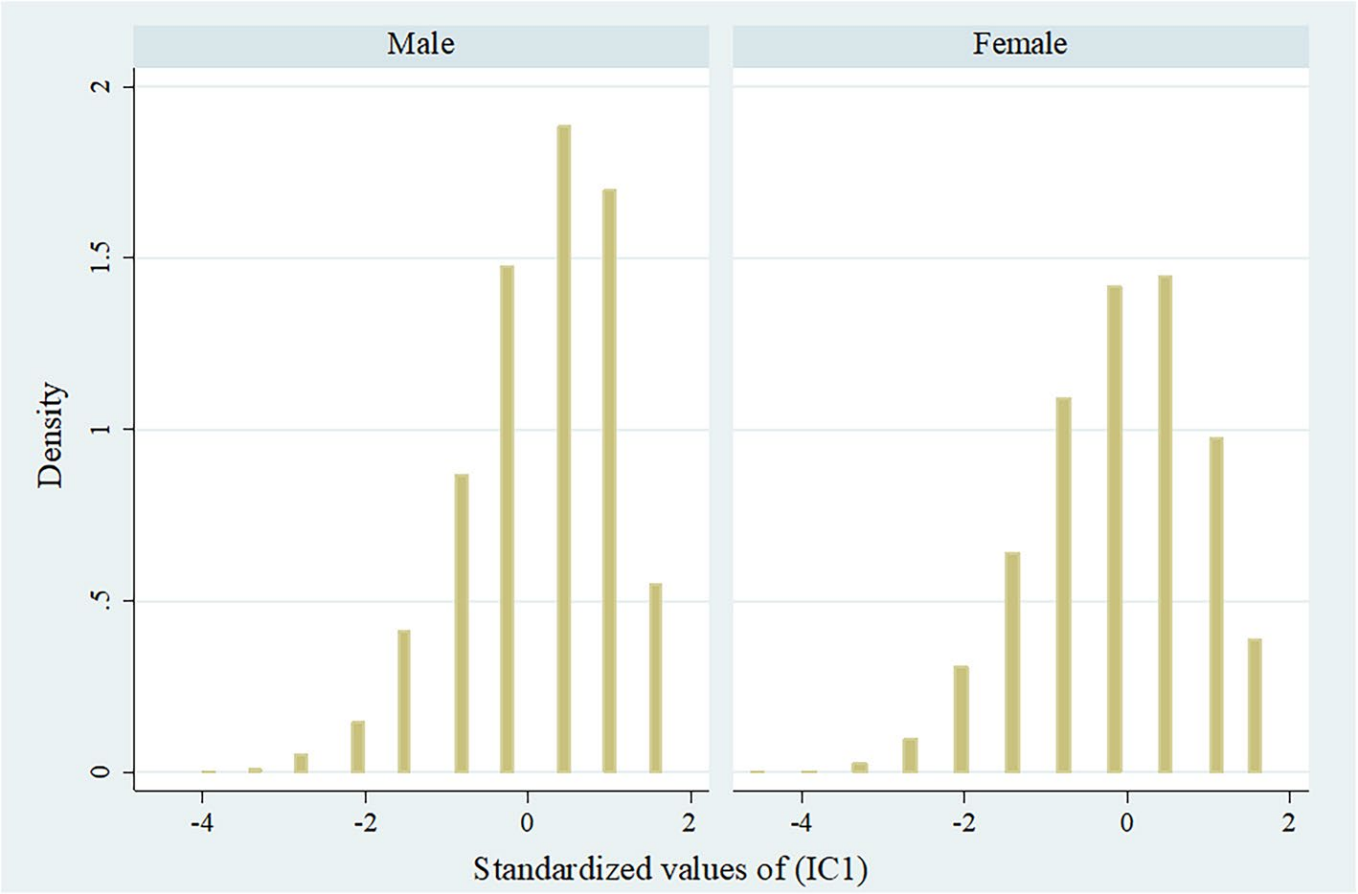
Distribution of intrinsic capacity composite z-score for the older men and women sample (histogram)

**Fig. 2:**
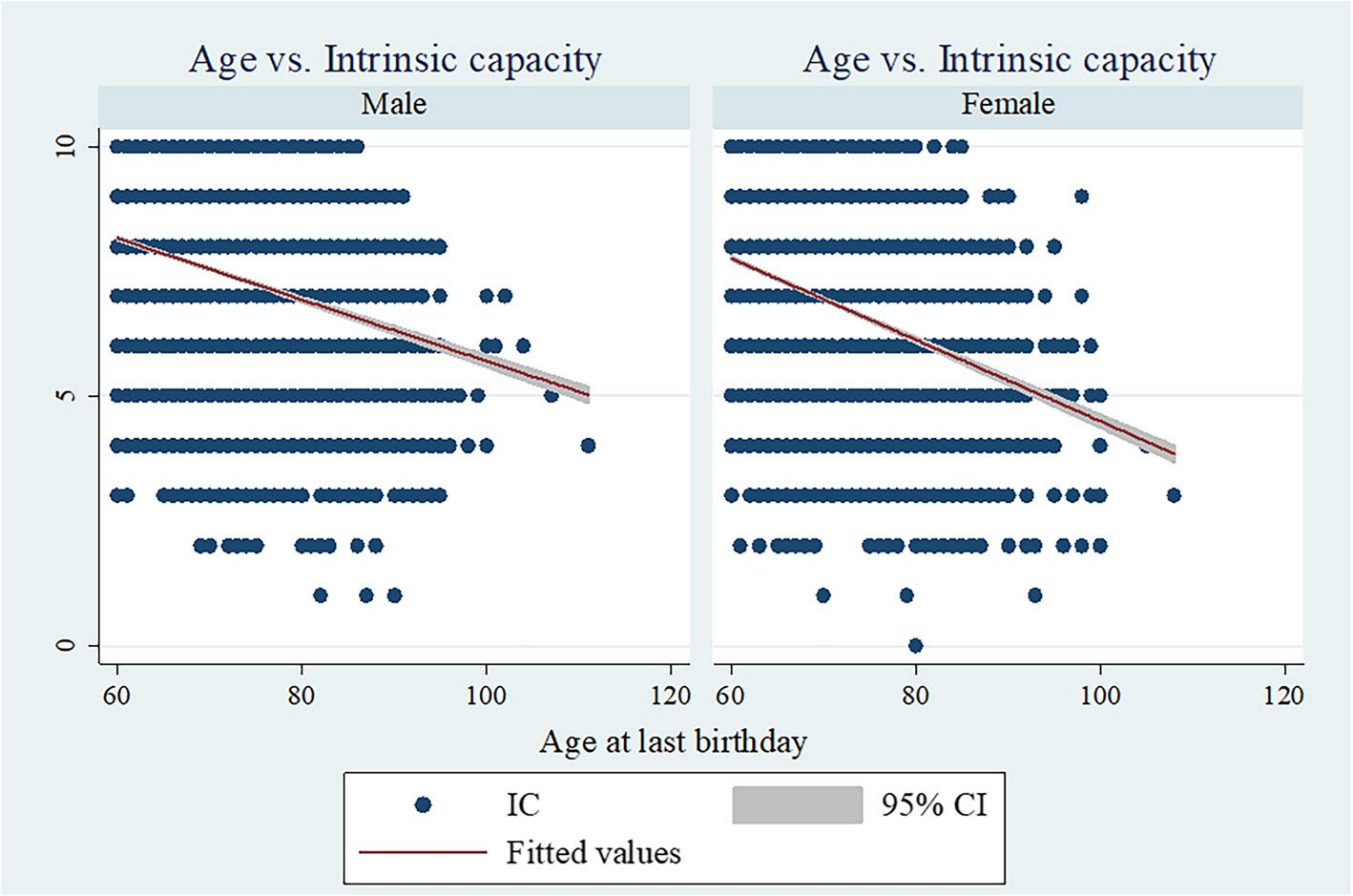
Scatter plot showing the correlation between intrinsic capacity scores and age. Pearson’s correlation coefficient was -0.287 on 11,871 male observations (95% CI: -0.303 to -0.270) and -0.340 on 12,265 female observations (95% CI: -0.355 to -0.324), which means negative correlations between IC and age among older men and women

The composite z-score of IC ranged from -4·62 to 1·65 in the study sample. Figure 1 shows the distribution of the IC composite z-score for male and female samples (histogram). Further, Fig. 2 presents the scatter plot of the age of the respondents and IC. There was a negative correlation between IC and older age among males and females (Pearson's correlation coefficient = -0.287, 95% CI: -0.303 to -0.270 among males; and -0.340, 95% CI: -0.355 to -0.324 among females).

Table 2 shows the descriptive statistics of study participants by background characteristics. The study estimated a mean IC score of 7.37 (SD = 1.6). The proportion of the sample constituted by older men and women aged 80 plus years was 9.47 and 8.14%, respectively. 37.41% of older men and 71.25% of older women had no formal education. Of the lifestyle factors, among older men, 27.88% smoked tobacco, and 32.5% chewed tobacco; among older women, it was 3.9 and 18.19%, respectively. 10.06% of older men and 1.1% of older women reported episodic alcohol drinking. 13.23 and 43.74% of older men engaged in moderate and vigorous physical activity, respectively, whereas 7.64 and 24.21% of older women engaged in moderate and vigorous physical activity. About 17.73% of older men and 12.32% of older women engaged in the yoga-related activity. A large share of older men and women were in the union and rural residents.

**Table 2:** Sample distribution of older adults by background characteristics

**Variables** (#missing cases)	Total		Male	Female
	Frequency	w col%	w col%	w col%
**IC**	Mean = 7.37	SD = 1.60		
**Age (in years)**				
60–69	15,408	62.30	61.11	63.41
70–79	6,752	28.92	29.41	28.45
80 +	1,976	8.79	9.47	8.14
**Sex**				
Male	11,871	48.51		
Female	12,265	51.49		
**Marital status**				
Currently in union	15,844	63.75	81.69	46.84
Widowed	7,692	34.18	15.92	51.39
Others	600	2.07	2.38	1.77
**Educational status**				
None	12,489	54.83	37.41	71.25
Primary	4,563	17.80	22.53	13.33
Secondary	4,994	19.21	26.82	12.04
Higher	2,090	8.16	13.23	3.38
**Work status**				
Never worked	6,575	25.92	3.53	47.02
Not working	8,017	33.92	37.55	30.5
Working	7,424	32.66	45.42	20.63
Retired	2,120	7.50	13.5	1.85
**Smoke tobacco** (#7)				
No	20,125	84.46	72.12	96.1
Yes	4,004	15.54	27.88	3.9
**Chew tobacco** (#7)				
No	18,653	74.87	67.5	81.81
Yes	5,476	25.13	32.5	18.19
**Episodic alcohol drinking** (#6)				
No	22,540	94.55	89.94	98.9
Yes	1,590	5.45	10.06	1.1
**Moderate physical activity** (#33)				
No	21,710	89.65	86.77	92.36
Yes	2,393	10.35	13.23	7.64
**Vigorous physical activity** (#5)				
No	16,179	66.31	56.26	75.79
Yes	7,952	33.69	43.74	24.21
**Yoga-related activity** (#13)				
No	20,197	85.06	82.27	87.68
Yes	3,926	14.94	17.73	12.32
**MPCE quintile**				
Poorest	4,833	20.02	20.81	22.3
Poorer	4,921	20.39	21.13	21.4
Middle	4,997	20.70	21.63	20.65
Richer	4,858	20.13	19.73	19.55
Richest	4,527	18.76	16.7	16.1
**Caste**				
SC/ST	7,761	26.40	26.39	26.42
OBC	9,306	46.14	45.92	46.36
Others	7,069	27.45	27.69	27.23
**Religion**				
Hindu	17,736	83.13	82.78	83.46
Muslim	2,852	10.73	11.13	10.35
Others	3,548	6.14	6.09	6.19
**Place of residence**				
Urban	8,183	28.61	26.37	30.73
Rural	15,953	71.39	73.63	69.27
**Region**				
North	4,496	12.72	12.56	12.86
Central	3,369	21.75	23.51	20.09
East	4,589	24.55	25.76	23.41
Northeast	2,901	2.95	3.02	2.89
South	5,758	22.14	20.13	24.04
West	3,023	15.89	15.03	16.71
**Total**	24,136	100		

*%* Percentage, *w col%* Weighted percentages to account for survey design and provide national population estimates, *SD* Standard deviation; High IC refers to older adults with a score of greater than mean IC plus one SD, *MPCE* Monthly per capita consumption expenditure

Concerning the prevalence of high IC (Table 3), older adults aged 60–69 had a higher prevalence of high IC (male 34.34% and female 24.6%) than their senior counterparts. Older adults who do not smoke tobacco (male 31.23% and female 21.13%) and do not chew tobacco (male 30.11% and female 22.44%) had a higher prevalence of high IC. Both older males and females reported episodic alcohol drinking had a higher prevalence of high IC (male 29.89% and female 20.65%). Older males and females who engage in vigorous physical activity (male 31.23% and female 21.07%) had a higher prevalence of high IC than those who do not engage in vigorous physical activity. But the result is not significant. Older men and women who engage in moderate physical activity (male 41.54% and female34.2%) and yoga-related activity (male 40.43% and female 29.94%) had a higher prevalence of high IC than their counterparts who do not engage in moderate physical activity and yoga related activity. People with higher levels of education and belonging to higher socioeconomic groups had a higher prevalence of high IC. Compared to participants, those excluded from this study due to incomplete data tended to be older, widowed and belonging to lower socioeconomic groups (Supplementary table S1).

**Table 3:** Prevalence of high IC among older adults by background characteristics

**Variables**	Total		Male		Female	
	w row%	*p*-value	w row%	*p*-value	w row%	*p*-value
**Age (in years)**		< 0.001		< 0.001		< 0.001
60–69	29.24		34.34		24.6	
70–79	18.30		21.99		14.7	
80 +	11.97		15.27		8.35	
**Sex**		< 0.001				
Male	28.90					
Female	20.46					
**Marital status**		< 0.001		< 0.001		< 0.001
Currently in union	28.41		31.01		24.15	
Widowed	17.63		18.94		17.25	
Others	20.22		23.35		16.26	
**Educational status**		< 0.001		< 0.001		< 0.001
None	12.00		12.82		11.6	
Primary	25.19		24.29		26.62	
Secondary	45.54		41.29		54.48	
Higher	58.13		57.12		61.86	
**Work status**		< 0.001		< 0.001		< 0.001
Never worked	25.54		19.15		25.99	
Not working	17.43		21.14		13.12	
Working	25.80		30.09		16.7	
Retired	47.98		48.71		42.9	
**Smoke tobacco**		< 0.001		< 0.001		< 0.001
No	25.31		31.23		21.13	
Yes	20.44		22.85		4.2	
**Chew tobacco**		< 0.001		< 0.001		< 0.001
No	25.80		30.11		22.44	
Yes	20.85		26.36		11.57	
**Episodic alcohol drinking**		0.008		< 0.001		< 0.001
No	24.91		29.89		20.65	
Yes	18.35		20.01		4.1	
**Moderate physical activity**		< 0.001		< 0.001		< 0.001
No	22.92		26.99		19.33	
Yes	38.75		41.54		34.2	
**Vigorous physical activity**		< 0.001		< 0.001		0.007
No	23.08		27.09		20.27	
Yes	27.47		31.23		21.07	
**Yoga-related activity**		< 0.001		< 0.001		< 0.001
No	22.55		26.43		19.12	
Yes	35.98		40.43		29.94	
**MPCE quintile**		< 0.001		< 0.001		< 0.001
Poorest	16.28		22.11		11.15	
Poorer	20.79		25.3		16.59	
Middle	25.50		30.66		20.4	
Richer	28.47		30.91		26.15	
Richest	34.45		37.28		31.69	
**Caste**		< 0.001		< 0.001		< 0.001
SC/ST	15.48		18.89		12.27	
OBC	26.26		29.65		23.09	
Others	30.43		37.2		23.93	
**Religion**		0.033		0.007		< 0.001
Hindu	24.82		29.17		20.76	
Muslim	21.59		28.12		14.96	
Others	26.19		26.74		25.68	
**Place of residence**		< 0.001		< 0.001		< 0.001
Urban	40.68		44.83		37.31	
Rural	18.10		23.2		12.99	
**Region**		< 0.001		< 0.001		< 0.001
North	24.55		31.16		18.46	
Central	15.62		19.21		11.66	
East	20.06		26.28		13.61	
Northeast	26.95		33.41		20.6	
South	31.61		32.78		30.69	
West	33.47		40.56		27.46	
**Total**	24.56					

*%* Percentage, *w row%* Weighted percentages to account for survey design and provide national population estimates, *SD* Standard deviation; *High IC* refers to older adults with a score of greater than mean IC plus one SD, *MPCE* Monthly per capita consumption expenditure

Table 4 provides multivariable linear regression estimates of IC by socioeconomic and lifestyle factors among older adults in India. For older men and women, increasing age was negatively associated with high IC. People in the age group 80 + were less likely to experience high IC (β = -1.11; CI: -1.29—-0.92) than their younger counterparts. Females were also less likely to experience high IC (β = -0.17; CI: -0.25—-0.08) compared to their male counterparts. Older adults with higher levels of education (β = 0.86; CI: 0.75—0.96) were more likely to experience high IC. With regard to lifestyle factors, people who smoke tobacco (β = -0.23; CI: -0.32—-0.13) and chew tobacco (β = -0.11; CI: -0.18—-0.03) were less likely to experience high IC compared to their respective counterparts. Older adults who reported episodic alcohol drinking was less likely to have high IC (β = -0.20; CI:-0.32—-0.07). The engagement in moderate physical activity (β = 0.12; CI:0.01–0.23), vigorous physical activity (β = 0.12; CI:0.05–0.20) and yoga-related activity (β = 0.18; CI:0.09–0.26) were significantly positively associated with high IC. Furthermore, older individuals who were widowed, residing in rural areas, belonging to households of SC/ST groups and the poorest wealth quintile were less likely to experience high IC.

**Table 4:** Multivariable linear regression estimates (adjusted coefficients) of IC by socioeconomic and lifestyle factors among older adults

Variables	Sub-categories	Total	Male	Female
		**aCoef. (95% CI)**	**aCoef. (95% CI)**	**aCoef. (95% CI)**
**Age**	60–69	Ref	Ref	Ref
	70–79	-0.46*** (-0.54—-0.38)	-0.38*** (-0.47—-0.30)	-0.53*** (-0.65—-0.41)
	80 +	-1.11*** (-1.29—-0.92)	-0.95*** (-1.20—-0.70)	-1.29*** (-1.54—-1.04)
**Sex**	Male	Ref	-	-
	Female	-0.17*** (-0.25—-0.08)	-	-
**Marital status**	Currently in union	Ref	Ref	Ref
	Widowed	-0.28*** (-0.37—-0.20)	-0.22*** (-0.34—-0.10)	-0.31*** (-0.42—-0.20)
	Others	-0.33*** (-0.50—-0.16)	-0.27** (-0.52—-0.03)	-0.40*** (-0.64—-0.16)
**Educational status**	No	Ref	Ref	Ref
	Primary	0.60*** (0.51—0.69)	0.57*** (0.44—0.70)	0.57*** (0.45—0.69)
	Secondary	0.97*** (0.87—1.08)	0.88*** (0.78—0.98)	1.09*** (0.88—1.30)
	Higher	1.12*** (1.01—1.23)	1.13*** (1.00—1.26)	1.15*** (0.98—1.33)
**Work status**	Never worked	Ref	Ref	Ref
	Not working	-0.21*** (-0.31—-0.11)	0.07 (-0.10—0.25)	-0.24*** (-0.35—-0.12)
	Working	0.12** (0.02—0.22)	0.43*** (0.26—0.61)	0.07 (-0.06—0.19)
	Retired	0.12* (-0.01—0.26)	0.46*** (0.27—0.65)	0.07 (-0.16—0.30)
**Smoke tobacco**	No	Ref	Ref	Ref
	Yes	-0.23*** (-0.32—-0.13)	-0.17*** (-0.28—-0.06)	-0.40*** (-0.56—-0.24)
**Chew tobacco**	No	Ref	Ref	Ref
	Yes	-0.11*** (-0.18—-0.03)	-0.07 (-0.17—0.03)	-0.17*** (-0.28—-0.06)
**Episodic alcohol**	No	Ref	Ref	Ref
	Yes	-0.20*** (-0.32—-0.07)	-0.19*** (-0.33—-0.06)	-0.07 (-0.36—0.21)
**Moderate physical activity**	No	Ref	Ref	Ref
	Yes	0.12** (0.01—0.23)	0.09 (-0.02—0.20)	0.18 (-0.03—0.39)
**Vigorous physical activity**	No	Ref	Ref	Ref
	Yes	0.12*** (0.05—0.20)	0.10** (0.01—0.18)	0.15** (0.03—0.27)
**Yoga-related activity**	No	Ref	Ref	Ref
	Yes	0.18*** (0.09—0.26)	0.19*** (0.10—0.29)	0.14* (-0.02—0.30)
**MPCE quintile**	Poorest	Ref	Ref	Ref
	Poorer	0.11** (0.01—0.21)	0.04 (-0.10—0.19)	0.15** (0.02—0.29)
	Middle	0.15*** (0.05—0.25)	0.09 (-0.02—0.21)	0.19** (0.04—0.35)
	Richer	0.22*** (0.12—0.31)	0.10 (-0.02—0.22)	0.30*** (0.16—0.43)
	Richest	0.25*** (0.14—0.37)	0.12* (-0.01—0.25)	0.36*** (0.20—0.53)
**Religion**	Hindu	Ref	Ref	Ref
	Muslim	-0.00 (-0.10—0.09)	0.09 (-0.03—0.22)	-0.10 (-0.24—0.03)
	Others	-0.00 (-0.11—0.11)	-0.06 (-0.23—0.10)	0.06 (-0.09—0.22)
**Caste**	SC/ST	Ref	Ref	Ref
	OBC	0.19*** (0.11—0.27)	0.22*** (0.11—0.33)	0.17*** (0.05—0.29)
	Others	0.19*** (0.10—0.27)	0.25*** (0.14—0.36)	0.13** (0.00—0.25)
**Place of residence**	Urban	Ref	Ref	Ref
	Rural	-0.43*** (-0.51—-0.36)	-0.33*** (-0.41—-0.25)	-0.48*** (-0.59—-0.36)
**Region**	North	Ref	Ref	Ref
	Central	-0.30*** (-0.39—-0.21)	-0.32*** (-0.43—-0.21)	-0.31*** (-0.44—-0.17)
	East	-0.27*** (-0.37—-0.18)	-0.26*** (-0.39—-0.12)	-0.31*** (-0.43—-0.18)
	Northeast	0.03 (-0.07—0.13)	0.04 (-0.08—0.17)	0.03 (-0.12—0.18)
	West	0.11** (0.01—0.21)	0.04 (-0.07—0.15)	0.14* (-0.01—0.30)
	South	0.06 (-0.03—0.16)	0.01 (-0.12—0.14)	0.11* (-0.02—0.25)
Constant		7.44*** (7.27—7.61)	7.09*** (6.84—7.34)	7.34*** (7.15—7.53)
Observation		24,077	11,837	12,240
Adjusted R2		0.2980	0.2766	0.2959

*aCoef* Regression coefficients adjusted for all the covariates, *MPCE* Monthly per capita consumption expenditure

^*^if *p*-value < 0.05

^**^ if *p*-value < 0.005

^***^ if *p*-value < 0.001

Table 5 shows adjusted odds ratios from logistic regression of each component of IC by socioeconomic and lifestyle factors among older adults. Among the five domains of IC, education was significantly associated with higher capacity in each domain, and increasing age was found to be a significant predictor of lower capacity in each IC domain except locomotion. Older females had lower odds of high capacity in each IC domain except the vitality domain. Older females had 73% (aOR = 1.73; CI: 1.47—2.04) significantly higher odds of higher capacity in the vitality domain. Sex difference in the psychological domain was not statistically significant. It was also revealed that older adults who reported episodic alcohol drinking had lower odds (aOR = 0.59; CI: 0.48—0.74) of high cognitive capacity. Older adults who engaged in yoga-related activity had 32% (aOR = 1.32; CI: 1.10—1.57) significantly higher odds of high cognitive capacity. It is also observed that older adults who smoked tobacco (aOR = 0.60; CI: 0.48—0.75), chewed tobacco (aOR = 0.82; CI: 0.71—0.96) and consumed alcohol (aOR = 0.68; CI: 0.54—0.86) had lower odds of high capacity in vitality domain. Also, older adults engaged in yoga-related activity had 25% (aOR = 1.25; CI: 1.07—1.47) significantly higher odds of having high capacity in the vitality domain. Engagement in vigorous physical activity is significantly associated with lower vitality capacity (aOR = 0.88; CI: 0.76—1.01). Interestingly, lifestyle factors had no significant impact on the locomotion domain. Older men and women engaged in vigorous physical activity had 35 and 19% significantly higher odds of high capacity in sensory (aOR = 1.35; CI: 1.12—1.62) and psychological (aOR = 1.19; CI: 1.06—1.34) domains, respectively. Other lifestyle factors had no significant association with the capacity of sensory and psychological domains.

**Table 5:** Adjusted odds ratios from logistic regression of each components of IC by socioeconomic and lifestyle factors among older adults

**Variables**		**High cognitive function**	**High vitality**	**Improved locomotion**	**Improved sensory**	**Improved psychology**
		aOR (95% CI)	aOR (95% CI)	aOR (95% CI)	aOR (95% CI)	aOR (95% CI)
**Age**	60–69	Ref	Ref	Ref	Ref	Ref
	70–79	0.69*** (0.60—0.80)	0.63*** (0.53—0.74)	0.98 (0.86—1.11)	0.46*** (0.40—0.53)	0.69*** (0.59—0.81)
	80 +	0.51*** (0.40—0.64)	0.50*** (0.33—0.75)	1.08 (0.89—1.31)	0.18*** (0.14—0.22)	0.46*** (0.38—0.56)
**Sex**	Male	Ref	Ref	Ref	Ref	Ref
	Female	0.57*** (0.49—0.65)	1.73*** (1.47—2.04)	0.87** (0.76—0.98)	0.64*** (0.54—0.75)	0.92 (0.80—1.06)
**Marital status**	Currently in union	Ref	Ref	Ref	Ref	Ref
	Widowed	0.88* (0.77—1.01)	0.87* (0.74—1.01)	0.72*** (0.64—0.82)	0.79*** (0.68—0.91)	0.91 (0.80—1.04)
	Others	0.73** (0.56—0.97)	0.58*** (0.41—0.83)	0.55*** (0.42—0.72)	1.04 (0.74—1.45)	0.99 (0.73—1.34)
**Education**	No	Ref	Ref	Ref	Ref	Ref
	Primary	3.05*** (2.66—3.49)	1.62*** (1.36—1.93)	1.08 (0.95—1.23)	1.09 (0.93—1.29)	1.03 (0.90—1.19)
	Secondary	7.43*** (6.14—8.99)	2.13*** (1.72—2.63)	1.30** (1.05—1.60)	1.56*** (1.19—2.04)	1.19 (0.92—1.53)
	Higher	14.60*** (11.17—19.08)	2.33*** (1.81—3.00)	1.37** (1.07—1.75)	1.40** (1.06—1.85)	1.72*** (1.36—2.16)
**Work status**	Never worked	Ref	Ref	Ref	Ref	Ref
	Not working	1.03 (0.87—1.22)	0.76*** (0.64—0.90)	0.89 (0.77—1.04)	0.77*** (0.65—0.90)	0.76*** (0.64—0.91)
	Working	1.15 (0.96—1.38)	0.70*** (0.58—0.85)	1.07 (0.91—1.25)	2.17*** (1.76—2.68)	1.15 (0.96—1.36)
	Retired	1.22 (0.94—1.58)	0.93 (0.72—1.21)	1.22* (0.96—1.55)	1.00 (0.74—1.35)	0.84 (0.64—1.10)
**Smoke tobacco**	No	Ref	Ref	Ref	Ref	Ref
	Yes	0.89 (0.75—1.04)	0.60*** (0.48—0.75)	0.95 (0.84—1.09)	0.98 (0.82—1.17)	0.89 (0.77—1.03)
**Chew tobacco**	No	Ref	Ref	Ref	Ref	Ref
	Yes	0.90 (0.80—1.02)	0.82** (0.71—0.96)	0.97 (0.87—1.08)	1.01 (0.87—1.17)	1.05 (0.93—1.18)
**Episodic alcohol**	No	Ref	Ref	Ref	Ref	Ref
	Yes	0.59*** (0.48—0.74)	0.68*** (0.54—0.86)	1.06 (0.87—1.27)	0.92 (0.68—1.23)	1.03 (0.84—1.26)
**Moderate physical activity**	No	Ref	Ref	Ref	Ref	Ref
	Yes	1.02 (0.79—1.30)	1.11 (0.90—1.37)	0.91 (0.76—1.08)	1.25 (0.94—1.65)	1.17 (0.97—1.41)
**Vigorous physical activity**	No	Ref	Ref	Ref	Ref	Ref
	Yes	1.12 (0.97—1.31)	0.88* (0.76—1.01)	1.08 (0.97—1.21)	1.35*** (1.12—1.62)	1.19*** (1.06—1.34)
**Yoga-related activity**	No	Ref	Ref	Ref	Ref	Ref
	Yes	1.32*** (1.10—1.57)	1.25*** (1.07—1.47)	1.00 (0.88—1.13)	1.12 (0.90—1.39)	1.00 (0.88—1.15)
**MPCE quintile**	Poorest	Ref	Ref	Ref	Ref	Ref
	Poorer	1.10 (0.95—1.28)	1.33*** (1.08—1.63)	1.17** (1.03—1.33)	1.00 (0.85—1.19)	0.92 (0.80—1.06)
	Middle	1.21** (1.04—1.42)	1.51*** (1.24—1.85)	1.12 (0.97—1.30)	0.92 (0.76—1.11)	0.96 (0.83—1.12)
	Richer	1.29*** (1.09—1.52)	1.78*** (1.45—2.20)	0.96 (0.81—1.15)	1.04 (0.86—1.25)	0.94 (0.81—1.10)
	Richest	1.25** (1.02—1.54)	2.12*** (1.70—2.64)	1.09 (0.90—1.31)	0.98 (0.76—1.27)	0.77** (0.60—0.99)
**Religion**	Hindu	Ref	Ref	Ref	Ref	Ref
	Muslim	1.15* (0.98—1.35)	1.05 (0.87—1.28)	1.02 (0.89—1.17)	0.83** (0.70—0.98)	0.90 (0.78—1.04)
	Others	0.97 (0.81—1.15)	1.13 (0.94—1.37)	1.30*** (1.10—1.53)	0.90 (0.74—1.10)	0.71*** (0.59—0.86)
**Caste**	SC/ST	Ref	Ref	Ref	Ref	Ref
	OBC	1.16** (1.01—1.33)	1.27*** (1.07—1.51)	1.25*** (1.11—1.40)	0.95 (0.82—1.11)	1.11* (0.98—1.26)
	Others	1.15* (0.99—1.34)	1.47*** (1.23—1.75)	1.04 (0.91—1.19)	0.88 (0.74—1.05)	1.01 (0.87—1.19)
**Place of residence**	Urban	Ref	Ref	Ref	Ref	Ref
	Rural	0.61*** (0.53—0.71)	0.48*** (0.42—0.55)	0.98 (0.86—1.12)	1.10 (0.95—1.28)	0.85** (0.73—0.98)
**Region**	North	Ref	Ref	Ref	Ref	Ref
	Central	1.20** (1.02—1.41)	0.53*** (0.44—0.65)	0.68*** (0.60—0.78)	0.90 (0.75—1.06)	0.83** (0.72—0.97)
	East	1.07 (0.92—1.25)	0.52*** (0.43—0.62)	0.88** (0.78—1.00)	1.06 (0.89—1.27)	0.74*** (0.65—0.85)
	Northeast	1.27*** (1.08—1.50)	0.46*** (0.37—0.57)	1.84*** (1.56—2.18)	1.10 (0.91—1.33)	0.99 (0.84—1.16)
	West	1.60*** (1.33—1.92)	1.30*** (1.09—1.55)	0.77*** (0.65—0.90)	0.80** (0.66—0.97)	1.06 (0.89—1.26)
	South	0.78*** (0.66—0.92)	0.97 (0.81—1.15)	1.15* (0.98—1.34)	1.32*** (1.09—1.60)	1.60*** (1.34—1.90)
Constant		0.61*** (0.46—0.79)	0.28*** (0.21—0.39)	1.45*** (1.13—1.85)	6.24*** (4.70—8.29)	3.61*** (2.83—4.60)
Observation		24,077	24,077	24,077	24,077	24,077

*aOR* OR adjusted for all the covariates, *MPCE* Monthly per capita consumption expenditure

^*^ if *p*-value < 0.05

^**^ if *p*-value < 0.005

^***^ if *p*-value < 0.001

## Discussion

The present study aimed to understand the prevalence of higher IC among older adults and to explore the socio-demographic and lifestyle factors, including tobacco use, episodic alcohol consumption and physical activity, associated with IC by analysing a large sample of older adults in India. This population-based study observed a mean IC score of 7.37 on a scale of 1–10, which was consistent with a recent study reporting that, on average, the Indian older population is slightly healthier, with a mean Healthy Ageing Index of 81.4 out of 100 [28]. The study analysing the status of perceived quality of life of older adults in India also highlighted that the overall quality of life of older people in India ranged from good to excellent even though there is a variation on the basis of socioeconomic characteristics [29].

On the contrary, the finding suggests that only a tiny proportion of older adults (24.56%) were enjoying higher IC. Studies conducted in LMICs also reported the higher prevalence of declining ICs in those country contexts [11, 13, 30]. The study shows a negative relationship between increasing age and IC among older adults, which is consistent with the result of a recent study on IC in the Chinese context [14]. This is also in line with earlier findings that as people get older, they are more likely to have functional decline and neurological or degenerative chronic conditions [31–33]. It was also observed that increasing age was a significant predictor of lower capacity in each IC domain except psychology, in concordance with the finding of recent studies conducted among Indian older adults, which found that as age increased, functional ability and psychological well-being decreased [34–36].

The findings based on the sex-stratified analysis underlines the gender gap in IC scores, with female older adults having lower IC scores than their male counterparts, which is consistent with the previous research finding [11]. The gender gap may root in differences in chronic conditions and the complex interplay between behavioural, pathological and environmental factors. In addition, females reported lower capacity in all sub-domains of IC except sensory. No significant gender gap was observed in sensory domain. Female disadvantages can be explained on the basis of ‘sensitivity theory’, which proposes that men and women are susceptible to the effects of health-related variables in different ways and ‘exposure theory’, which hypothesises a disparity in exposure to health determinants between men and women [37]. Generally, it is believed that men live shorter but healthier lives than women, who live longer but are in poorer health [38]. Also, growing literature has revealed that women have greater overall rates of physical disease, more disability days, and more doctor visits, hospital stays despite having lower mortality rates at practically every age than men do [38].

The present study further highlights that currently being in a marital union and living arrangement with spouse and children act as a protective factor against IC decline and is consistent with the findings of earlier studies that stressed on marriage as protection against adverse health outcomes through changing health behaviours and social networks originating from the union [39–41]. Multiple studies in demographic research have found that married people had better health and lower mortality than unmarried people [42, 43]. Previous studies in the Indian context with cross-sectional nature have also established the significance of marital status on health outcomes and emphasised that widowed status is linked to poorer mental and physical health [44–46]. With regard to living arrangements, it was already demonstrated that living with a spouse only and solitary living were closely associated with short-term morbidity [47].

Furthermore, findings of our study highlight the importance of maintaining healthy lifestyle behaviours for improving late-life functional capacity. The study found that engagement in moderate or vigorous physical activity acts as a catalyst for higher IC among older people. The positive impact of physical activity on IC was already confirmed in previous studies [14, 16, 19]. Physical activity was found to be the most effective preventative intervention in the treatment of cognitive dysfunction, physical frailty, disability, and risk of falls and fractures [30, 48]. Notably, the findings suggest that yoga-related activities are positively associated with IC among older Indian adults. In a narrative review on physical activity and mental health in India, exercise and yoga were found to be useful in lowering mean scores for both severe and common mental disorders, and yoga had a greater impact on schizophrenia patients than either exercise or no intervention [49].

It is also evident from the multivariate analysis that health risk behaviours such as tobacco use and episodic alcohol consumption adversely affected the IC score of the study population. This evidence was consistent with the earlier study findings that healthy lifestyle behaviours have been linked to improved health outcomes in older adults [20]. In the Indian context, it was already verified that older adults who smoked tobacco and who consumed alcohol had a considerably increased risk of having cognitive damage [50] and a higher prevalence of multimorbidity [51]. With regard to the effect of smoking, an indirect relationship between smoking and body weight has been observed in studies [52, 53]. The review analysing the consequences of cigarette smoking and nicotine on the behavioural, sensory, and metabolic systems of the human body explained the mechanism by which smoking may alter weight [54]. As documented, paying attention to one's lifestyle, such as food, exercise, not smoking or drinking excessively, and remaining mentally active, may be the most efficient way to healthy ageing [1, 22].

The social gradient in IC is evident from the analysis. That is, lower IC scores were associated with lower educational attainment or a lower economic condition. Consistent with the literature on IC, the study observed that people with secondary or higher education were more likely to report higher IC scores than their counterparts with primary or no education [8, 9, 11, 55, 56]. The connection between increasing educational attainment and improved health is well documented across different disciplines [57]. A possible justification for the relationship between higher educational level and higher IC score can be given by the ‘brain reserve hypothesis’, which states that more years of education implies more cognitive capacity [58, 59]. The finding also verifies the empirical evidence from the Indian studies on the positive effects of higher education on quality of life and physical health of older people [60, 61]. Moreover, education was found to be a significant predictor of higher capacity in each sub-domain of IC. Further, our analysis underlined the strong positive association of economic status in terms of wealth quintiles and work status with higher IC. The direct association of economic status with IC has also been observed in previous studies [11, 14].

Regarding work status, higher IC was reported for people who were retired or currently working. Studies exploring the link between labour market participation and IC are scarce in the literature. This needs to be further investigated. The study also found that people residing in rural areas were more likely to report lower IC compared to urban people. This rural disadvantage corroborates the findings of earlier studies on the rural–urban disparity in resource availability, quality of life, health perception, physical and psychological health and successful aging among older adults in India [62–65]. The reason for rural–urban differentials in health-related variables can be rooted in their lower education and lower economic status [66]. There is a shortage of studies investigating the connection between IC and place of residence as well as regional variations. Future studies can be directed towards these aspects.

### Implications of the current findings

The present study has implications in societies where population ageing is taking place, and healthy ageing policies are a major concern. As the current study suggested, priority must be given to the oldest-old, female, illiterate and rural residing people who are more vulnerable to lower IC when formulating healthy ageing policies. The study also implies that focusing on sex-specific strategies would be highly effective in geriatric health interventions. In addition, to enhance IC and thereby optimise functional abilities in late life, modifiable risk factors such as tobacco use, alcohol consumption and physical inactivity should be targeted. Governement should implement measures such as higher taxation on tobacco products and alcohol and an advertisement ban for intimidating use of such substances.

Also, it would be highly beneficial if older people were made aware of the importance of engaging in regular physical activity and exercise to stay healthy and productive. Promotion of yoga and exercise, encouragement to join small walking groups, organisation of fitness classes for senior citizens etc. can be effective moves in this respect. Moreover, the provision of nutrition supplements, the inclusion of fruits and vegetables in daily diets etc., can also be emphasised in healthy lifestyle behaviours, which are associated with better health outcomes and an increase in the number of years spent in good health [20, 21].

### Limitations and strengths

Our study has certain limitations. Firstly, sensory and psychological domains were measured using self-reported indicators, which may adhere to reporting and recall biases. Secondly, due to the cross-sectional nature of data, it is difficult to sufficiently ascertain the observed relationships between IC and its predictors. In order to overcome this limitation, future studies of longitudinal nature are recommended. Thirdly, we have excluded certain components like grip strength and sleep disturbance in IC construction. In future studies, IC can be modified, including such components. Importantly, the current analysis dropped a proportion of the sample due to incomplete information on IC components. The significance tests of differences in the included and excluded sample showed that the missing cases include people from higher age group, females, widowed and those who are socioeconomically poor. This might negatively influene the generalizability of the current findings. Also, the cut-off point for higher IC as one standard deviation from the mean IC score should be investigated further and validated in specific socio-cultural settings. Lastly, even though we have included maximum potential confounders in our study, there are still some context-specific confounders like health-seeking behaviour and health-related variables like multimorbidity and perceived health status out of our consideration. Such factors should be taken into account in further studies.

Despite these limitations, the study is unique in several aspects. Firstly, it is the pioneer study assessing IC among older adults in the Indian context. Secondly, the study specifically explored the impact of lifestyle behaviours on IC. Only a few studies are available examining the association of lifestyle factors with IC [14, 23, 67]. Thirdly, the study carefully accounted for the impact of maximum potential confounders, including work participation and place of residence, which are ignored in most of the studies. Fourthly, most of the components of IC domains except sensory and psychological domains are either performance-based measures or anthropometric measures, which are free from reporting and recalling biases. Lastly and most importantly, our study is based on a large nationally representative sample of older adults in India which allow the generalizability of the findings.

## Conclusion

The study revealed that lifestyle behaviours, including tobacco use, episodic alcohol drinking and physical activity, are strongly associated with IC among older adults in India. The findings have more significant implications for geriatric health care and healthy ageing policy formulation in India and other LMICs with comparable demographic and socio-economic transitional phases. The current findings also suggest that healthy lifestyle behaviours should be encouraged among older adults to improve their IC, which is the crucial determinant of functional abilities and quality of life in later years of life.

## Supplementary Information

**Additional file 1: Table S1. **Differences in socio-demographic characteristics of included and excluded sample in the study. **Table S2.** VIF estimates for the selected explanatory variables. **Figure S1.** Kernel density plot showing the normality of residuals.

## Data Availability

The datasets generated and analysed during the current study are available in the IIPS data repository and accessible on reasonable request through The administrative permission was obtained from the International Institute for Population Sciences, Mumbai for using the data for the current analysis.
