# Supplementary material for: Socio-demographic and lifestyle factors associated with intrinsic capacity among older adults: evidence from India
Source: BMC Geriatr. 2022 Nov 12;22:851. doi: 10.1186/s12877-022-03558-7 (PMC9652958; doi:10.1186/s12877-022-03558-7)
Supplement: Supplementary file 1 — Additional file 1: Table S1. Differences in socio-demographic characteristics of included and excluded sample in the study. Table S2. VIF estimates for the selected explanatory variables. Figure S1. Kernel density plot showing the normality of residuals. [file 12877_2022_3558_MOESM1_ESM.pdf]

Supplemental material

Table S1: Differences in socio-demographic characteristics of included and excluded sample in the study

Variables	Included (n=24,136)	Excluded due to incomplete data (n=7,328)	Difference	Chi2 p-value
Age				<0.001
	62.3	48.66	13.64	
	28.92	32.06	-3.14	
	8.79	19.28	-10.49	
Sex				<0.001
	48.51	44.04	4.47	
	51.49	55.96	-4.47	
Marital status				<0.001
	63.75	55.62	8.13	
	34.18	41.31	-7.13	
	2.07	3.07	-1	
Living arrangement				<0.001
	5.75	5.17	0.58	
	20.06	19.15	0.91	
	43.11	35.59	7.52	
	31.07	40.09	-9.02	
Education				<0.001
	72.63	77.47	-4.84	
	19.21	15.17	4.04	
	8.16	7.36	0.8	
Work status				<0.001
	25.92	30.14	-4.22	
	33.92	40.57	-6.65	
	32.66	21.47	11.19	
	7.5	7.82	-0.32	
MPCE quintile				<0.001
	20.02	22.53	-2.51	
	20.39	21.23	-0.84	
	20.7	19.36	1.34	
	20.13	17.9	2.23	
	18.76	18.97	-0.21	
Caste status				<0.001
	26.4	34.83	-8.43	

	46.14	35.21	10.93	
	27.45	29.97	-2.52	
Religion				0.097
	83.13	72.34	10.79	
	10.73	12	-1.27	
	6.14	15.67	-9.53	
Place of residence				0.123
	28.61	34.88	-6.27	
	71.39	65.12	6.27	
Regions				<0.001
	12.72	17.96	-5.24	
	21.75	12.19	9.56	
	24.55	15.94	8.61	
	2.95	11.61	-8.66	
	22.14	24.84	-2.7	
	15.89	17.47	-1.58	

MPCE: Monthly per capita consumption expenditure

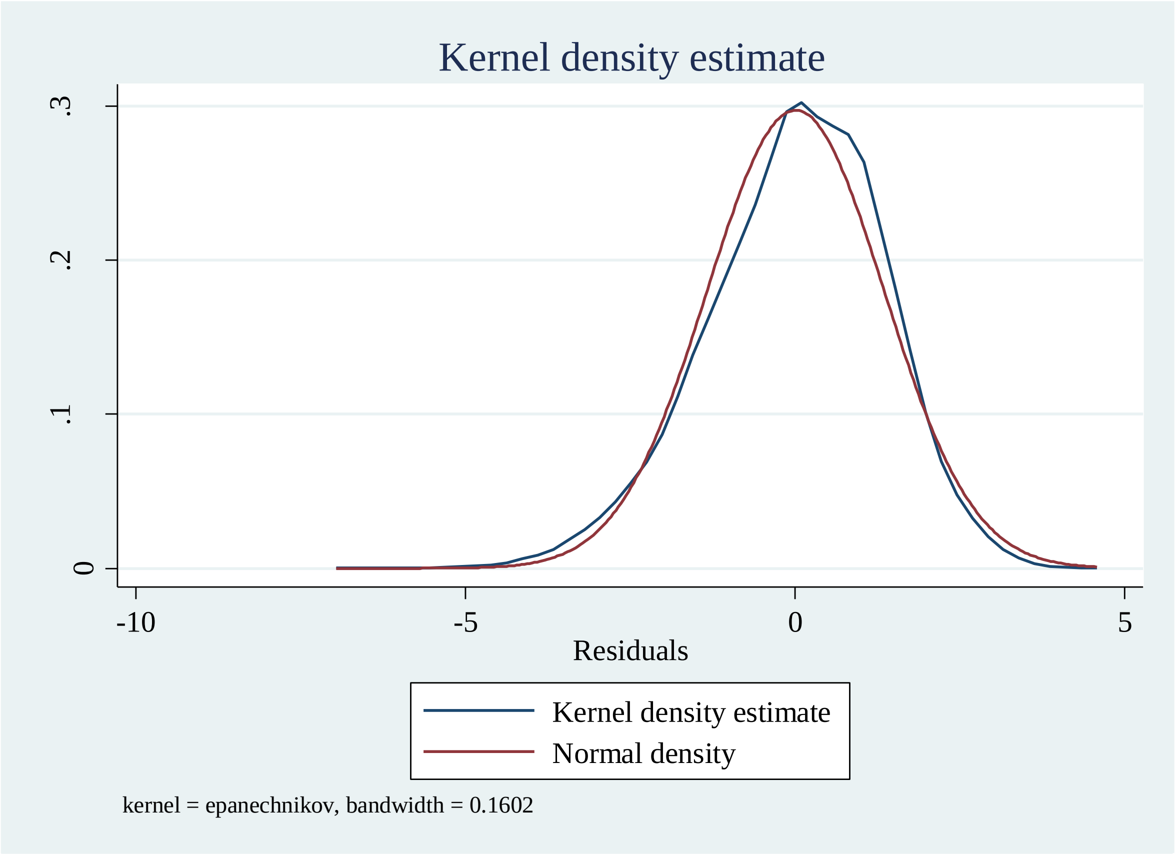

Figure S1: Kernel density plot showing the normality of residuals

Table S2: VIF estimates for the selected explanatory variables

Variables	Sub-categories	VIF	1/VIF
**Age**	60-69		
	70-79	1.13	0.882813
	80+	1.16	0.860372
**Sex**	Male		
	Female	2.08	0.480371
**Marital status**	Currently in union		
	Widowed	1.3	0.769613
	Others	1.02	0.976605
**Educational status**	No		
	Primary	1.22	0.816984
	Secondary	1.47	0.681755
	Higher	1.62	0.616247
**Work status**	Never worked		
	Not working	1.97	0.508335
	Working	2.48	0.402723
	Retired	1.62	0.616117
**Smoke tobacco**	No		
	Yes	1.34	0.745309
**Chew tobacco**	No		
	Yes	1.3	0.766597
**Episodic alcohol**	No		
	Yes	1.1	0.909277
**Moderate physical activity**	No		
	Yes	1.17	0.85491
**Vigorous physical activity**	No		
	Yes	1.34	0.746554
**Yoga-related activity**	No		
	Yes	1.19	0.842217
**MPCE quintile**	Poorest		
	Poorer	1.58	0.6321
	Middle	1.62	0.619148
	Richer	1.62	0.615864
	Richest	1.64	0.61142
**Religion**	Hindu		
	Muslim	1.1	0.905111
	Others	1.11	0.901879
**Caste**	SC/ST		
	OBC	1.71	0.583286
	Others	1.81	0.552774
**Place of residence**	Urban		
	Rural	1.33	0.754115
**Region**	North		

	Central	2.31	0.432528
	East	2.46	0.406006
	Northeast	1.24	0.805888
	West	2.48	0.40334
	South	2.08	0.480153
Mean VIF		1.57	

VIF: Variance inflation factor; MPCE: Monthly per capita consumption expenditure
